# The operating theatre as a classroom: a literature review of medical student learning in the theatre environment

**DOI:** 10.5116/ijme.5ca7.afd1

**Published:** 2019-04-23

**Authors:** Stefanie M. Croghan, Catherine Phillips, William Howson

**Affiliations:** 1 University College London, Postgraduate Department of Medical Sciences, UCL Medical School, UK

**Keywords:** Learning in the operating theatre, teaching in the operating theatre, operating room education, student experiences of surgery, surgical education for medical students

## Abstract

**Objectives:**

We set out to review the published literature relating to the educational experiences of medical students in the operating theatre.  In particular, we wished to deduce from the current evidence what challenges are posed to student learning in this environment, and how they may be overcome.

**Methods:**

National Library of Medicine and Google Scholar databases were searched from 1990-2018, using search terms ‘Operating Theatre,’ OR ‘Operating Theater,’ OR ‘Operating Room’ AND ‘Medical Students.’ Title and abstract review of 679 papers were performed.  Full-text English language papers about the learning or satisfaction of medical students in the theatre environment were included.  Papers exploring the experiences of residents/trainees rather than medical students were excluded.  A total of 36 papers were eligible for inclusion.  Thematic analysis was conducted on these papers.

**Results:**

A number of common themes were identified.  Throughout the literature, medical students describe a lack of clear learning objectives, fear, anxiety, feelings of humiliation and intimidation, lack of visualisation and lack of opportunity for participation as barriers to their satisfaction with theatre placements and to their subjective learning.

**Conclusions:**

Obstacles identified by students as deleterious to their experiences in the operating theatre are remarkably reproducible across a number of research studies in different populations.  Areas to address by both individual educators and curriculum designers include fostering a culture of inclusion in theatre, setting explicit, achievable learning goals for students in this environment and making a concerted effort to prepare students for the theatre setting.

## Introduction

Surgical exposure is incorporated into the curriculum of virtually all medical schools, and widely considered a necessary component of the undergraduate experience.  Resultingly, each year thousands of medical students make their first foray into a ‘classroom’ which bears little resemblance to any they have experienced before – the operating theatre.  Simultaneously, medical educators adopt the role of the teacher in this unique environment.   We were keen to explore the variables influencing learning in this setting, with a view to identifying how education may be optimized.

As a learning environment, operating theatres have many resources to be exploited.   Changing the teaching domain (e.g. from classroom to outdoors) is recognised within education as potentially beneficial.^1^ Thus, introducing the medical student to the novel theatre environment may incite interest and heighten the senses.  Theatre naturally offers multimodal stimuli, and consequently is inviting to students of all learning styles: visual, auditory, read/write and kinaesthetic.^2^ The traditional aim of improving clinical/exam-related performance is addressed, but students also gain an understanding of surgical specialities, learn about “surgical culture,” and may be inspired in future career choice.^3^^, ^^4^ “Going to theatre gives [students] a better understanding of surgery than [they] get just reading the textbooks.”^5^

However, there are two sides to any coin.  Just as the unique facets of the operating theatre foster a rich and powerful learning experience, so too do the intricacies of theatre present medical students with a number of challenges to contend with.  The hierarchical operating theatre can be a stressful environment, with intimidation a common theme reported by medical students and theatre staff alike.^6^  There is an enshrined but unwritten code of conduct, and students are challenged to “pick up cues on the run,”^5^ whilst overcoming personal emotions and fears.^7^  Perhaps most importantly, the operating theatre is real.  Removed from the relative safety of the classroom environment, students are relocated in a fast-paced, unpredictable world, where complications arise and mistakes have consequences.  Their usual source of guidance, the tutor, is now a surgeon at work in the professional environment, with the tutor role secondary to operative performance and patient care.  This reduces the tutor’s capacity to use referential and ego-supportive skills identified as important in the education literature.^8^ Certainly, this environment may enrich the learning experience, fostering independent learning and personal growth, but so too may the surgeon’s domain present a challenge to theatre-naïve medical students.

We wished to explore the subjective educational benefit of operating theatre attendance for medical students, and the factors influencing this.  We found a lack of integrated research in the current literature in this area.  While a variety of heterogeneous studies on a local level had been conducted, we found it was difficult to infer global relevance or generalisability to our own practice from the results.  We, therefore, proposed to perform a systematic review, integrating the current evidence and ascertaining which, if any, findings recurred throughout the published literature and appeared to remain applicable outside of the local context.

Our specific objectives were to discover, according to the currently published evidence: i How medical students perceive their time in theatre, ii. What challenges medical students face in the theatre that may influence the educational experience, iii. How such challenges may be overcome and how educators can facilitate this.  By becoming more cognisant of the obstacles faced by medical students, we propose that eduators can work at both altering the milieu to make it more inviting, and at equipping students with the skills necessary to navigate the surgical environment.

## Methods

A critical narrative approach was used in this literature review, with the intent of systematically identifying and synthesizing the published literature related to the learning of medical students in the operating theatre environment.

### Search process

Literature search strategies were designed for the National Library of Medicine (NLM) and Scopus databases, employing search terms ‘Operating Theater’ OR ‘Operating Room’ AND ‘Medical Students’.  A search of the afore-mentioned databases was conducted by the first author (SC) and reviewed by the senior author (WH).   Title and abstract review of all papers produced by the search strategy were performed.  In addition, the reference list of included papers was reviewed to identify any further papers eligible for inclusion.

### Article selection

**Figure 1:**
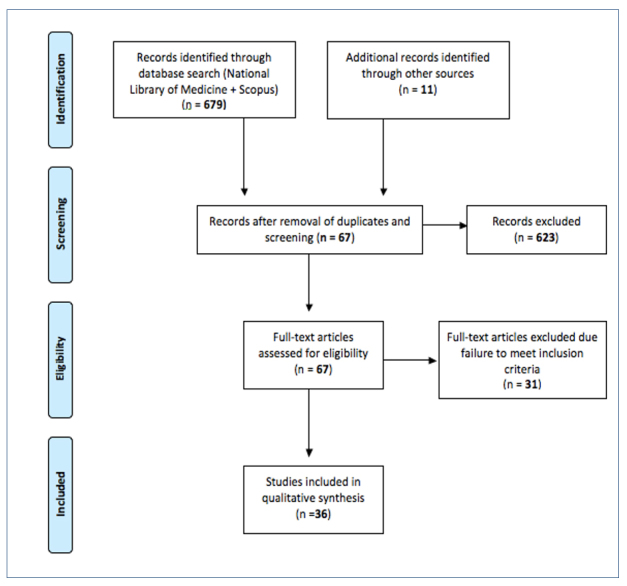
Article Selection Process

Inclusion criteria were research papers published 1990-2018 pertaining to the learning or satisfaction of undergraduate/postgraduate medical students in the theatre environment and written in the English language.  Opinion pieces written from the perspective of educators only, papers relating to the experiences of postgraduate surgical trainees/residents and published abstracts without associated full-text articles were excluded.   A total of 679 papers were identified from the search results.  Following removal of duplicates, screening according to inclusion and exclusion criteria, and additional hand trawl of the reference list, 36 papers were included.  The article selection process is outlined in a flow diagram in Figure 1.^9^

### Data synthesis and analysis

Papers were reviewed.  Data extracted included study characteristics (year of publication, country of origin), participant information (number of participants, year of study, rotation at the time of the study) research methods and findings. Thematic analysis^10^ of each paper’s results was performed by the first author (SC) and reviewed by the other two authors.  A multi-phase sequential approach to analysis was applied.  The findings of each paper were reviewed, with manual coding applied, using a spreadsheet.  Codes were reviewed by paper and grouped into themes.  Papers were then reviewed synchronously, with themes refined and integrated to capture findings that emerged across the literature.  All differings of opinion between the authors were agreed by discussion.   A number of common factors emerged throughout the literature, and these are discussed by theme in the remainder of this paper.

**Table 1:** The primary sources

Author	Year	Country	Population	Research Method	Themes Identified
Lyon^5^	2003	Australia	Medical students and surgeons	Mixed Methods - Group interviews (medical students) - In-depth semi-structured interviews (students, n=15 and surgeons, n=10) - Questionnaire (n=197, response - rate 87%)	Learning goals unclear for medical students
					Fear in strange environment may hamper learning
					Embarrassment / fear of looking foolish experienced by students Time versus educational benefit of theatre
					attendance questioned by students
Ravindra^33^	2013	UK	Recently qualified medical school graduates	Questionnaire (n=209, 67% response rate)	Learning goals unclear for medical students
					Victimisation and humiliation experienced by students
					Students desire active participation / to ‘scrub in’
					Time versus educational benefit of theatre attendance questioned by students
Zundel^11^	2015	Germany	Medical students from year three upwards Surgeons	Series of focus groups (medical students, n=17 and surgeons, n=10)	Common themes identified included: Learning goals unclear for medical students Teaching strategy of faculty influences learning Fear in a strange environment may hamper learning
Fernando^13^	2007	Scotland	Final year medical students	Questionnaire (medical students, n=54 response rate 90%)	Learning goals unclear for medical students
					Feeling welcome important to students
Fernando^12^	2007	Scotland	Final year medical students Consultant trainers	Questionnaire (medical students, n=46 100% response rate; consultants, n=42 46% response rate)	Learning goals unclear / discordant
					Lack of visualisation an issue for students
Irani^14^	2010	US	Medical students and faculty	Mixed – field observations and satisfaction ratings. Assessing amount and type of teaching in the operating theatre, relative to curriculum goals. Student n=11.	Learning goals unclear / discordant
					Students may not require active
					participation to have a positive experience
O’Neill^15^	2017	US	Third-year medical students, attending surgeons, surgical residents	Questionnaire answered by 57 total participants: Medical students, n= 25 (43.8% response rate) of those who have completed their third-year surgical clerkship, n= 14 (24.6% response rate) of those who have not completed their third-year surgical clerkship, attending surgeons, n=9 (15.8% response rate), surgical residents, n = 9 (15.8% response rate)	Learning goals unclear / discordant
					Feeling burdensome an issue for students
Hampton^4^	2011	US	Fourth-year medical students on Obstetrics & Gynecology clerkship and faculty	Focus groups (two focus groups including 13 medical students, one focus group including five faculty members)	Learning goals more aligned students/ faculty in this study
					Practical learning a relevant goal in view of some faculty
					Welcomeness / integration into team recognised as important by faculty
Flannery^16^	2014	Northern Ireland	Third-year medical students completing a neurosurgery placement	Questionnaire (n=22, 8% response rate of all students, however not all were eligible as had not attended neurosurgery theatre)	Preparedness of students important
					Learning goals (can be) unclear (or lack a degree of clarity) for students
					Teaching strategy/style of faculty influences learning
Lee^17^	2005	Scotland	Fourth-year medical students following ENT placement	Questionnaire (n=152, response rate 100%)	Learning goals sometimes unclear / discordant / not achieved
					Lack of visualisation an issue for students objectives
					Hampton^18^	2014	US	Medical students on Obstetrics & Gynaecology clerkships	Pre and post intervention questionnaire (n=68 completed post-clerkship and n=27 completed at six months post-clerkship, of a group of 70)	The benefit of clearly stipulated learning objectives
										Positive opinion of faculty teaching correlated with high satisfaction overall
Hubbell^19^	1996	US	Medical students	Questionnaire, medical student n=48 (98% response rate)	The benefit of setting clear learning objectives
					Teaching strategies - the role of visual reinforcement
Callcut^21^	2004	US	Surgical faculty and medical students	70+/- 7 student evaluations of 74 academic surgeons	Teaching strategy/style of faculty influences learning
Bowrey^7^	2014	UK	Third and fourth-year medical students on a perioperative care placement	Semi-structured interviews (n=9 of 83 invited students)	Fear in a strange environment may hamper learning
					Intimidation experienced by students
					Feeling welcome, team integration important to students
Morzycki^24^	2016	Canada	Medical students of all years	Questionnaire (n=180, response rate 40%)	Fear in a strange environment may hamper learning
					Intimidation experienced by students
					Teaching strategies – the benefit of preparatory course
Chapman^25^	2013	UK	Medical students of all years	Questionnaire (n=292, response rate 20.8%)	Feeling welcome, team integration important to students
					Active participation important to students
Stone^23^	2015	Canada	Final year medical students and recent graduates	Questionnaire (n=72, response rate 21%)	Fear in a strange environment may hamper learning
					Intimidation experienced by students
					Teaching strategies – the benefit of preparatory course anticipated by students
Miandoab^26^	2016	Iran	Medical students in semester 4 and semester 8	Questionnaire (n=62)	Feeling welcome, team integration important to students
Lyon^27^	2004	Australia	Final year medical students	Mixed Methods - Group interviews (medical students) - In-depth semi-structured interviews (students, n=15 and surgeons, n=10) - Questionnaire (n = 197, response rate 83%)	Humiliation
					Feeling welcome, team integration important to students
					Active participation important to students
					Teaching strategy/style of faculty influences learning
Thomas^28^	2006	UK	Final year medical student	Personal reflection	Humiliation
					Teaching strategies: benefit of a preparatory (simulated operating theatre) course
Pettitt^29^	2004	US	Third-year medical students	Questionnaire (n = 84, response rate 83%)	Fear in a strange environment may hamper learning
					Mistreatment experienced by students
Coveney^32^	2013	Ireland	Third and fourth-year medical students	Free recall experimental model, assessing recall in two different learning environments	The learning of medical students as assessed by the short-term recall can be preserved in a variety of environments
Knight^34^	2017	UK	Penultimate year medical students who had just completed their neurosurgical placement	Questionnaire (n =201, response rate 81.4%)	Feeling welcome, team integration important to students
					Students perceive theatre exposure as useful
Cloyd^35^	2008	US	First-year medical students involved in a ‘Surgical Skills Elective.’	Implementation of Surgical Skills elective followed by questionnaire (n=55 questionnaire responses from 30 students, response rate 88.7%)	Feeling welcome, team integration important to students Feeling burdensome common amongst medical students Teaching strategies – the benefit of a surgical skills workshop
					Active participation important to students
Hong^38^	1996	Canada	Fourth-year medical students on surgical clerkship	Implementation of computer-based tutorials on human anatomy before theatre attendance. Evaluated by questionnaire (n= eight medical students and an additional questionnaire completed by faculty also)	The benefit of setting clear learning objectives
					Teaching strategies – the benefit of a preparatory anatomy course
Patel^39^	2013	US	First and second-year medical students	Medical students enrolled in an introductory workshop ‘Surgical Saturday’ and completed pre and post-workshop questionnaires (n=33)	Students lack confidence regarding operating theatre etiquette and behaviour
					Teaching strategies – the benefit of a preparatory workshop
Patel^40^	2012	UK	First-year medical students	Intervention - students were randomized into four groups for operating theatre preparation: control, didactic lecture, ‘second life,’ and simulated operating suite. Participants completed a pre and post intervention questionnaire. N=60	Teaching strategies – the benefit of a preparatory workshop
Martin^41^	2012	UK	Medical students	A workshop for medical students was designed based on responses of medical students (n=36) and consultant surgeons (n=8) to a questionnaire. A workshop was then delivered to 147 medical students and feedback collected by questionnaire.	Teaching strategies – the benefit of a preparatory workshop
					Students lack confidence in the operating theatre environment
Drolet^42^	2014	US	Pre-clinical medical students	Implementation of a preclinical elective in surgery, using a paired resident-mentorship model. Student exposure and confidence with clinical activities evaluated by questionnaire before and after the elective (N = 24, 100% response rate).	Teaching strategies – the benefit of a preparatory course
					Students lack confidence in the operating theatre environment
Shipper^43^	2018	US	Pre-clinical medical students	Implementation of a technical and nontechnical skills curriculum, evaluated by semi-structured interviews of students (n=8) and instructors (n=5).	Teaching strategies – the benefit of a preparatory course
					Fear in a strange environment may hamper learning
					Intimidation experienced by students
Broderick^44^	2002	US	Variety of theatre staff	The trial of an endoscopic video camera and telescope attached to an operating table, with common objects placed on the operating table in mock surgical fields. Persons (n=11) from a variety of medical backgrounds evaluated the images on the adequacy of visualization.	Lack of visualisation an issue for students
Berman^45^	2008	US	Third-year medical students	Questionnaire (n=116, response rate 89%) following a surgical clerkship, during which a structured mentorship programme was in place.	Teaching strategies – the benefit of a preparatory course
					Active participation important to students
					Teaching strategy of faculty influences learning
Schwind^46^	2004	US	Medical students	Questionnaire (completed for 114 learning episodes in the operating room)	Active participation – may not be important to students
					Teaching strategy of faculty influences learning
					Time versus educational benefit of theatre attendance questioned by students
Stark^47^	2003	UK	Fourth-year medical students and consultant clinical teachers	Focus groups of medical students (n=20 total) and semi-structured interviews of consultants (n=13)	Teaching strategy of faculty influences learning
					Time versus educational benefit of theatre attendance questioned by students
McIntyre^50^	2008	US	Third-year medical students on surgical clerkship	The pilot of teleconferencing sessions (live broadcasting of procedures to a classroom setting where students were based along with a faculty member). Observation performed of students (by educators) in operating theatre and the teleconference setting (n=23 observations) and questionnaires completed by students (n=78)	Teaching strategies – the benefit of a novel approach to intra-operative teaching
					Teaching strategy of faculty influences learning
					Time versus educational benefit of theatre attendance questioned by students
Jensen^20^	2018	Denmark	4^th^-year medical students enrolled in an undergraduate surgical introduction initiative involving assisting in the operating room with a surgical mentor (senior surgeon)	Ethnographic observation (n=7 students, 70 hours of observation)	Learning goals – hidden curriculum exists
					Students may lack confidence in the operating theatre environment
					Teaching strategies – the benefit of a novel approach to intra-operative teaching
					Teaching strategy of faculty influences learning

## Results

Thirty-six papers were included and underwent full-text analysis.

### Study characteristics

The primary sources identified and discussed in the remainder of this paper are summarized in Table 1. A number of themes emerged from the literature as relevant to the educational experience of theatre sessions for students.

### Learning goals

One issue reported by medical students is a lack of clarity regarding the purpose of their theatre placement.  A study by Lyon and colleagues found less than 50% of students to agree with the statement that “the objectives for attending theatre are clear to me.”^5^ The authors elicited this by a multi-modal qualitative approach, incorporating observation, student interviews, and related questionnaires, likely capturing students’ true opinions.  While one might wonder if these findings are outdated, the sentiment of confusion regarding learning objectives appears rather static. Indeed, some ten years later, Ravindra and colleagues found just 47% respondents “knew what was expected of them” in theatre in a questionnaire study of a broad demographic population, with a good (67%) response rate.   A mere 13% of students in this study had ‘set learning objectives,’ and probingly, this was correlated with reduced attendance. While the target of “attendance” is open to confounding factors, it seems an interesting and pertinent secondary outcome as attendance “sign off” was not performed in this institution.   Zundel and colleagues, in a series of focus groups, found that “students of all groups stated that they beneﬁted from being told about the learning objectives” and furthermore found that “insecurity about [learning] objectives led to [students] being less able to sustain knowledge.”^11^

Perhaps unsurprisingly, other studies demonstrate that when medical students are left ‘rudderless,’ without clear guidance as to educational goals, they may formulate learning objectives disharmonious with their seniors’ expectations.  Fernando and colleagues found 39% of a student group lacking instruction was “inappropriately overambitious” in aspirations to memorise “skills [and] techniques” “considered postgraduate-level knowledge.”^12^ The same authors showed students often endeavoured to describe operations and identify surgical instruments, while the majority of consultants felt these goals were “not applicable.”^13^ This study design captured the perspective of both medical students and consultant trainers by dissemination of parallel questionnaires, hence highlighting this discrepancy.

The emphasis of theatre conversation, however, may not always be congruent with surgical educators’ views of desirable learning goals and outcomes for medical students. One study observing medical students on surgical clerkship/placement noted that 55% of the intraoperative time was “spent teaching technical aspects of the operation.”^14^  Whilst very valid reasons may underlie this, including the presence of postgraduate surgical trainees in the operating theatre and a desire to stimulate students interested in pursuing a surgical career, it surely also will lead students to deduce that the learning emphasis is placed on memorizing technical details.

Similarly, O’Neill and colleagues, in a questionnaire-based study of medical students, consultant surgeons and residents identified a discrepancy in envisaged learning, with the majority of surgeons surveyed in agreement that medical decision-making, disease processes, and gross pathology were thoroughly taught and emphasized intraoperatively, whilst the majority of medical students disagreed with the same statement.^15^ Medical students in this study seemed of the opinion that learning technical skills was an integral objective to their time in the operating theatre, with 84% desiring more time devoted to this aspect, in sharp contrast to consultant surgeons, of whom only 11% felt more focus should be skills based.  Hampton and colleagues, in a focus group study of students on an obstetrics and gynaecology placement, along with surgeons, found student-surgeon goals more closely aligned.^4^ Some key differences, however, also emerged.  Faculty and students agreed that developing a foundation of clinical knowledge was an integral part of the operating room experience.  However, faculty stressed that “students should review and master baseline knowledge before entering the operating room,” emphasising that “it is more about reinforcement in the operating room.”  In contrast to O’Neill’s findings, faculty in Hampton’s study agreed that “teaching technique and skill was appropriate for several portions of an operating room experience,” and expressed that this may allow “students to incorporate into the team more.”^4^

Specialty-specific studies of students on neurosurgery^16^ and ENT^17^ placements have also revealed relatively low proportions (68.18% and 59.9%, respectively) of students reporting that their subjective learning goals have been met, although consultants’ perceptions are not explored for comparison in these studies.

Hampton and colleagues trialled a new curriculum for students (n=70) on obstetrics and gynaecology placement, and compared students were experiencing it to a control group on conventional clerkship.^18^ Integral to the intervention curriculum was clearly stipulated learning objectives and computer-based instructional content to help these be achieved.  The intervention group on post-clerkship questionnaire gave significantly higher evaluation scores for quality of faculty teaching and demonstrated increased retention of acquired clinical knowledge at 6 months.  Hubbell and colleagues, identifying anatomy as a key learning goal in operating room attendance, enhanced “instruction in regional gross anatomy” with “a series of illustrations projected in the operating room and viewed sequentially during major operations.”^19^ Such a method, as well as supporting the learning of anatomy, reinforces the learning objectives themselves. Medical student feedback revealed students viewed this method as efficacious.

Evidently medical students require formal guidance if they are to understand the aim of theatre sessions and correctly interpret learning priorities. It may be, however, that desirable learning goals are sometimes more abstract than medical students may conceptualize, and here a so-called hidden curriculum may appear.  Jensen and colleagues, using ethnographic observation, explore surgical education from the perspective of practice theory.^20^  The authors note the need for students to adapt to cultural-discursive arrangements within the operating room, where “time pressure calls for the use of slang…” and “the mix of professions and traditions creates new expressions…” Learning around relatings and “social-political arrangements” becomes paramount for a student to navigate the surgical environment successfully.  As such, a student is compelled to consolidate “sayings and doings of the surgical practice,” as well as to learn “relationships, norms and values” which may have been “articulated in a less systematic manner” at medical school.  It seems therefore quite possible, that some learning in theatre is not focused on mere factual recall.  Students, however, may not always grasp this perspective and may feel disenfranchised if they do not come away from a theatre session with a collection of acquired facts or demonstrable skill.  Potentially, describing the wider purpose of theatre placement to students could reorientate expectations and improve satisfaction.

### Teaching of faculty

Several studies show that students draw conclusions on surgeons’ interest in teaching and style of teaching while in the theatre environment, and often correlate this with their learning experience.  “Lack of interest” of the surgeon in teaching was cited by several students as a barrier to their learning in the neurosurgical theatre in a study by Flannery and colleagues based on questionnaire and semi-structured interview responses.^16^ Callcut and colleagues obtained 70+/- 7 student evaluations of 74 academic surgeons practising at a US medical school over a 4 year period.^21^  Results show that the more junior group of surgeons was “perceived by medical students to be more effective educators in the operating room” (1.79 ± 0.02 vs. 2.01 ± 0.01, p < .001).

Further exploration is needed to uncover the reasons that one cohort emerged as superior teachers in the eyes of the medical students, and the generalisability of this finding is unclear.  The study does, however, suggest a certain consistency and reliability in medical student assessment of an operating room educator, implying that teaching style is a significant variable in this context. Whilst students are vulnerable to feeling intimidated in the operating theatre^7^^,^^22^^,^^23^ Zundel and colleagues found students to positively view “being questioned,” as long as this was conducted in a “well-structured, non-malicious” way.^11^  An area of concern identified in Zundel and colleagues’ focus group studies was “a lack of certainty” on the part of students on which surgeon in the theatre “was in charge of the teaching.”  The authors also explored this issue with surgeons, and discovered both that “the issue of who was responsible for teaching was hardly ever raised,” and that “younger surgeons...felt unsure” about directing teaching where the senior surgeon operating did not carry it out, as they feared “being regarded as overconfident.”^11^  It seems clear that an agreement made by operating surgeons regarding teaching roles prior to a case beginning, and communication of teaching roles to participating medical students could ameliorate this.

### Emotional aspects

A myriad of emotions experienced in the theatre setting is reported by students throughout the literature, and their potential effect on the learning experience should be considered.

### Fear and intimidation

Fear is an emotion frequently described by students in relation to theatre placements.  Fear of syncope or of “violating protocol” is common.^7^^,^^24^ This appears heightened by a lack of knowledge with regard to “etiquette,” and confusion as to simple logistics, such as “where to stand” and “which doors to enter.”^12^^, ^^25^  Similarly, fears of accidentally acting in a way that “could adversely affect the patient,” for example through inadvertent contamination of a sterile object, arise.^5^  A survey based on student issues identified in a focus group was distributed to final year medical students and recent graduates by Stone and colleagues.^23^  Respondents (n=72) described a prevalent fear of “appearing incompetent” (89%), with an overwhelming majority (96%) attesting to being nervous when attending the operating theatre.  This appears a globally recognised phenomenon.  Zundel and colleagues in a focus group of medical students in Germany noted that “students recalled feeling intimidated by the [operating room] in general” and describing “feeling insecure regarding how to behave,”^11^ and Miandoab and colleagues^26^ in a questionnaire-based study of medical students in Iran (n=62) found a positive correlation between “a positive ethical climate” and educational attitude (p=0.03).

### Embarrassment

Humiliation is also common.  “Experience[ing] embarrassment” in the theatre was reported by 70% of students in a study by Lyon and colleagues.^27^ Validity of this finding is increased by the high (83%) response rate to the questionnaire, which was distributed to the substantial number of 237 students.  Similarly, another study revealed 70% of students surveyed felt “it’s easy to be made look a fool in theatre,”^5^ whilst the personal reflection of a medical student on his early days in theatre includes an incident of inadvertently desterilising an instrument trolley culminating in a public “chastising chorus of surgeon and scrub nurse.”^28^

### Anxiety

Perhaps unsurprisingly, anxiety is reported alongside fear, apprehension and humiliation.  Pettitt and colleagues surveyed a group of medical students prior to surgical clerkship and enquired about their expectations.^29^ The 84 respondents revealed significant anxiety regarding potential “poor personal performance,” “fatigue” and “mental abuse.” The precise effect of emotions on learning is not established in the operating theatre context.  Deleterious effects of stress on learning and cognition have been demonstrated, however,^30^ suggesting the Yerkes-Dodson law of arousal/stress beyond a threshold level having a negative effect on performance holds true.^31^  This view is corroborated by the revelation that two-thirds of students in a series of semi-structured interviews (n=9) reported experiencing anxiety “so overwhelming it was detrimental to learning” in theatre.^7^  Measuring learning itself is a difficult endpoint, but the student’s own perception is surely very relevant.  It is superficially countered by Coveney and colleagues who demonstrate no difference in memorisation/recall between theatre and tutorial room environments in a free recall experimental study of fourteen medical students.^32^  However, however, the validity of this study is disputable as the “theatre environment” was artificially simulated without the inherent stresses of an operating room in reality.  It seems that the fostering of a more encouraging atmosphere would be appreciated by students and may facilitate their learning.

### Feeling welcome

A study published in 2013 (n=209) demonstrated that students who reported being “made to feel welcome” were more likely to attend theatre opportunities than students who refuted this statement.^33^  Similarly, “feeling part of the team” was found to be a powerful determinant of student enjoyment in theatre, in a study focused on the emotional experiences of students over a 6-week surgical rotation elicited via in-depth interviews (n=9).^7^  It seems an unfortunate fact, therefore, that another study (n=54) found only 7% of medical students in theatre to “[feel] welcome all of the time.”^12^  These studies do not explore what specific features students identify as creating an atmosphere of welcomeness per se.  However, it is worth noting that Fernando and colleagues reported 74% of students (n=54) to rank “friendliness & approachability” of theatre staff as the most important factor in assisting their learning in this environment.^13^  Knight  and colleagues in a questionnaire-based study of UK medical students on a neurosurgical rotation (n=201) found that a positive 78.6% reported that they felt welcome in theatre, possible correlated with 67.7% of students in this study either agreeing or strongly agreeing that “attending at least one neurosurgical theatre session was beneficial.”^34^ It appears that as educators, focusing efforts on fostering a feeling of inclusion for medical students may enhance their theatre experience.

### Feeling burdensome

Students also report feeling like a “burden” or “nuisance.”^13^  Feeling unwelcome to the extent that “you often get a surgeon who doesn’t want you there” was declared by one student in the research by Chapman and colleagues.^25^  Cloyd and colleagues investigated students’ (n=36) views of their role in theatre, as compared to the perception of the students held by theatre staff.^35^  It is interesting to note that the students rated their contribution to the theatre team far lower than did surgeons and nurses present during the case.  Similarly, O’Neill and colleagues noted that while most surgeons and residents (55% and 66% respectively) agreed/strongly agreed that the surgical team benefitted from medical student presence, the students did not view themselves as such an asset.^15^  In fact, almost half of medical students (48%) felt their participation was not beneficial and commented on fears of “being in the way” or “bothering the surgical team with questions.” Emphasising the students’ potential to be useful within the environment may increase their comfort and likelihood of engaging.^36^

### Victimisation

Several papers elicit reports of students being unacceptably treated, in a theatre environment made hostile by consultant surgeons, trainees/residents or nursing staff.  While some recall bias may exist, and not all experiences may have been specifically theatre-based, Chapman and colleagues’ survey of alumni reflecting on their surgical clerkship experience identified mistreatment as a memory of some respondents.^37^ One alumus recalled that residents “made life unnecessarily harsh” for students.  Similarly, Stone and colleagues received student reports of being “mocked for the entire OR duration,” “laughed at,” being “hit because [of] getting too close to the sterile field” and fearing “getting called out in front of everyone,” to the extent that one student described theatre as a “very unsafe and hostile learning environment.”^23^  Ravindra and colleagues report that 48% (n= 209) of recently qualified doctors recalled receiving negative “put-downs” or “gibes” during theatre placements as students.^33^  This study notes that female doctors were more likely to report these than their male counterparts; however the possible reporting bias influencing this is not explored.

Whilst there is no evidence to suggest such encounters predominate, the effect of such experiences on the recipient student and others present must be considered.

### Preparedness

Some of the emotional aspects, for example the sense of apprehension and fear may be partially attributed to inadequate induction sessions. Champan and colleagues in a questionnare based study of 234 medical students noted ‘lack of preparedness’ to be a chief factor in unsatisfactory theatre experiences,^25^ and in a study of syncope/presyncope amongst 180 medical students, Morzycki and colleagues discovered a lecture on operating theatre etiquette (i.e. personnel, instruments used, and flow of theatre proceedings) was desired by many students prior to placement.^24^ Zundel and colleagues reported that focus group students “felt a general displeasure if they did not know what they were going to do and see the next day”, and felt that “personal and emotional preparation was not possible” if students were uninformed regarding “the duration of a procedure, the amount of blood involved, or the necessity for lead gowns.”^11^

The concept of preparedness can be addressed in both a specific and a general sense.  Locally, on a day-to-day or weekly basis, students may benefit from being advised on which theatre they will be assigned to and what cases will be taking place, to allow them to “read up on expected knowledge.”^11^  Hong and colleagues demonstrated the utility of “short, preoperative, computer-assisted tutorials on human anatomy” prior to general surgical cases and trialled with 8 medical students, finding that such an endeavour “can have a positive impact on the clerk's level of knowledge and confidence in the operating room.”^38^

In the pursuit of a more global introduction to surgery as a whole or to theatre itself, a number of authors have piloted various induction sessions.  Patel et. al designed a Saturday workshop of surgical induction / skills training attended by 33 junior (1st and 2nd year) medical students, primarily with the goal of increasing interest in surgical careers, however noted strong agreement that the session was helpful in making students feel better prepared for the surgical rotation or sub-internship, presumably assuaging some of the fear of the unknown.^39^ Stone and colleagues enquired whether a formal training session or workshop prior to surgical clerkship would make students more comfortable; only 5.6% (4/71) of students disagreed.^23^ A personal reflection by a medical student who had completed simulated operating theatre (SOT) exercises on an extracurricular basis reveals the preparation for real life practice such exercises can provide in a non-threatening environment.^28^ Furthermore, the potential of SOT to enhance inter-professional relations within the operating theatre by providing medical students with an insight into the roles of non-medical staff comes to light.  A further study of a pilot theatre preparation course, experimenting with the use of didactic lectures, a virtual theatre accessed via a computer lab and a replicated theatre, found all three approaches to result in significant improvements in students’ (n=60) knowledge, behaviour and attitudes related to the theatre environment.^40^   Martin and colleagues designed a preparatory course, based on results of a local questionnaire guiding the authors regarding deficient domains in theatre induction as perceived by medical students.^41^  A workshop was then delivered to 147 students, followed by evaluation.  Student feedback was extremely positive, with the majority of respondents agreeing that “they found the workshop useful,” that “it increased their confidence” and that they would recommend it to their peers.^41^  Drolet and colleagues at Brown University describes the merits of a mentorship based pre-clinical elective, trialled with 24 medical students.  The authors found the preparation of the elective to result in “considerable improvements in conﬁdence with observing” in the operating room and with operating room etiquette, as well as with scrubbing in and participating, as compared to control students.^42^ Shipper and colleagues from Stanford University piloted a pre-theatre course focused on delivering technical and non-technical relevant skills to 30 medical students, who were later evaluated with semi-structured interviews. A number of positive findings emerged, including the fact that “acquisition and synthesis of basic technical skills increase preclinical student comfort in the operating room.”^43^ While a selection bias may exist in recruitment to some such preparatory courses at the pilot stage, with surgically-inclined students availing of the opportunity, the reiteration of positive findings does suggest that greater preparation may help more students to avail of greater learning opportunities in the theatre setting, and merits consideration by curriculum designers.

### Visualisation

An intuitive variable affecting student enjoyment and learning in theatre is the visibility of the procedure.  Fernando and colleagues revealed 30% of students felt unable to visualise “much of the operation” and found this “detrimental.”^12^ The authors highlight the potential benefit of visual display units linked to a head camera worn by the surgeon.  The applicability of these finding is specialty-dependant. Teams performing endoscopic procedures, such as general, urological, thoracic and gynaceological specialists, and those who frequently utilise intraoperative imaging, like orthopaedics and vascular, may naturally grant students adequate visualisation.  For open surgeries, a head camera with a display unit as suggested by Fernando is beneficial.  Broderick and colleagues also describe the successful use of an endoscopic video camera and telescope attached to the operating table, which can project the image for student viewing.^44^  The utility of such approaches may be limited due resource constraints and variance in surgeons’ attitudes towards facilitation, but it is apparent that if student visualisation is neglected, a negative impact on enjoyment and sense of inclusion will result.

### Active participation

A further point of consideration is the influence on the student of his/her participation in surgery.  There are conflicting reports of the importance of the student being actively involved as an assistant.

Bermen and colleagues in a questionnaire study of 3rd-year medical students (n=116) found that students who had had the opportunity to suture, manage a laparoscopic camera, or otherwise “felt involved in the operating room” were more likely to be interested in surgery as a career (p ≤ 0.01).^45^ While this study did not look at the subjective educational benefit, one could hypothesize that this desire to enter a surgical field is reflective of student enjoyment of the theatre experience.  A study by Chapman and colleagues of 157 alumni asked to reflect on their surgical clerkship revealed “scrubbing in” to be cited as the most educationally beneficial activity experienced, by graduates who went on to pursue both surgical and non-surgical careers alike.^37^ In a survey-based study performed by Champan and colleagues at the University of Leeds, 73.6% (167/227) of undergraduates expressed a desire to scrub in more often.^25^ Ravindra  and colleagues (n=209) found students were more likely to attend theatre sessions if “invited” “to scrub” or allowed “active participation.”^33^  The validity of this finding is unclear, as there is no distinction between the actions.  Both incorporate the common factor of scrubbing in, which invariably equates to better visualisation and/or involvement in conversation.  It is difficult, however, to appreciate if acting as an assistant or performing surgical tasks offers additional benefit.  Other studies would suggest not.  A study performed by Irani and colleagues (n=11) showed that satisfaction was not influenced by operative involvement. This was reiterated by Schwind and colleagues who also concluded that active involvement did not influence the conduciveness of the theatre environment to students’ subjective learning, in a study of 114 experiences.^46^  There is strength in this finding as the authors extrapolated 27 variables potentially influencing learning, thereby triggering the students to evaluate them as independent factors.

Furthermore, surgical teams may be somewhat opposed to the concept of the student assistant.  One study that addressed trainer’s perceptions reported 43% of consultants studied felt students acting the second assistant was “not appropriate.”^12^ This is an interesting revelation. However, the precise implication is unclear.  Physicians selected this option on a standardised questionnaire where the adjacent option was “desirable”; arguably there lies a rather wide expanse of neutral territory between the two.

Regardless, from the available information, it seems the opportunity to ‘scrub in’ is correlated with improved student satisfaction, but that active student participation is less important.

### Time vs educational benefit

For medical students, time is at a premium.  Another factor influencing satisfaction with theatre placements is how worthwhile the student perceives the experience related to time expenditure.  Lyon and colleagues confirm students carefully “weigh the cost” of theatre time against other forms of learning which may facilitate the greater acquisition of knowledge “necessary to pass undergraduate exams.”^5^  Ravindra  and colleagues demonstrated a negative correlation between long operations and attendance.^33^  Stark  and colleagues in two focus group studies of medical students (n=20) similarly found a feeling that time spent in theatre was often not worthwhile; as one student commented, “Standing in on a surgical procedure for 4 hours, I’d say a lot of that is a waste of 4 hours…it’s absolutely pointless.”^47^ Hypothetically, students may have greater learning per unit time if assigned to short operations, which by their nature may also be associated with a less stressful environment and a greater percentage of total operating time dedicated to teaching.   For other cases, students benefit from “open communication” from the surgeon if he/she “envisages not being able to do much teaching,” in order to manage “expectations” and allow the student to make the best choice for his/her learning.^48^  An alternative learning experience for such cases may be the use of videos incorporated into classroom tutorials or lectures.^49^

One novel intervention is proposed by McIntyre and colleagues who trialled intraoperative education via teleconferencing (TC) with a group of third-year medical students (n=29).^50^ These students were placed in a classroom with a faculty member and watched live surgery via a video link with 2-way communication.  This is a unique scenario in that the faculty member’s sole purpose during the session is to teach, unlike other situations where an operating surgeon focused on a procedure and simultaneously trying to engage the students may experience role conflict. Students rated the TC educational experience significantly higher than the OR across all topics covered.  In the TC cohort, they felt more able to ask questions (p <0.01) and more felt it was a good use of their time (p =0.04).

## Discussion

The operating theatre possesses many unique factors distinguishing it from standard tutorial rooms, and as such, is a theoretically fascinating environment in which to teach and observe learning.   The response of the medical student to this foreign environment is, however, variable, and quite frequently negative.  Numerous articles outline the struggles of medical students to assimilate into operating room environments across the globe and across a number of decades.  In a 1986 publication, Folse and colleagues identified potential pitfalls of surgical placements, which the authors identified as “frequently unstructured” with “skill acquisition…left  largely to chance with little quality control,” “students inadequately monitored” and “feedback seldom given.”^51^ Jarringly, the same issues appear to prevail today.

### Implications for medical education

A number of factors emerge in this review as powerful influences on the medical student’s experience in the theatre environment.  Almost all are modifiable variables.  We propose that initiatives born out of liaison between curriculum designers, surgical teams and non-surgical members of the operating theatre staff could radically improve the student experience.  Pre-placement preparation strategies for students, with the establishment of agreed learning goals and some form of an introductory session to operating theatre etiquette and behaviour delivered in a non-threatening environment, appear to be positively embraced by students.  Such learning objectives should be set at the curriculum level and discussed with practising surgeons who supervise student placements.  Steps taken by educators to put in place specialty-specific measures ensuring adequate student visualisation of the operating field are crucial.  Sadly, it appears that medical students frequently perceive the theatre environment as hostile, and mistreatment is not uncommonly encountered.  It is quite clear that a concerted global effort to urgently address this situation is essential, in order to both optimise short-term educational benefit and to avoid potentially detrimental effects on the recruitment of students to surgical careers.  We feel that action is required by medical school authorities, departmental heads, individual surgeons (both consultant and trainee level) and nursing staff to ensure that the theatre environment is as welcoming as possible to students, whilst established priorities of patient safety and efficiency are maintained. Students’ satisfaction is linked with feelings of welcomeness and team integration, and this can be fostered by all members of the surgical team both inside and outside of the theatre environment.  It appears that many of the negative emotional experiences, based on fear, intimidation, embarrassment and anxiety, described by students on a level deleterious to their learning could be ameliorated by greater student and faculty preparation, along with a continued cultural shift towards a more inclusive and welcoming operating theatre ethos across the board.  We propose that this would enhance student learning within the operating theatre.

### Limitations

There is a reasonable body of literature addressing medical education within the theatre.  This includes a number of published papers highlighting obstacles identified by medical students as detrimental to their learning and enjoyment.  Most papers use students’ opinion as their measure.  While this may not equate to the most scientifically objective evidence, we feel it is a realistic and appropriate endpoint, as assessing a more distant target such as exam performance is open to such a degree of confounding bias that it is unfeasible. Current papers focus on students in individual universities.  This, of course, raises questions regarding generalisability of findings, with each student group a product of a particular curriculum and perhaps each affiliated teaching hospital harbouring a particular ethos.  Similarly, some studies are specialty-specific, while others incorporate students who have experienced a variety of different surgical specialties on placement yet evaluate their experience as a whole.  These are potential limitations.  However, the recurrent themes and remarkably similar opinions of a diverse, international selection of students reported throughout the literature does suggest global relevance.

### Areas for future research

There are of course areas which merit further research.  It would be useful to assess the optimal delivery of introductory sessions and/or the effect of a surgical mentor accompanying the students.  The current evidence consists of a variety of local pilot studies demonstrating the efficacy of various forms of induction.  However, multicentre trials with randomisation of medical students to different approaches and careful evaluation could provide useful information on effective and cost-friendly strategies to guide curriculum designers.  It would also be interesting to observe the outcomes of further research surrounding the application of novel forms of technology to enhance student visualisation.  The mini-surgical theatre educational environment measure developed and validated by Nagraj and colleagues may be used in future studies to assess the educational climate of the theatre as perceived by students and to measure change following implementation of an intervention.^52^

## Conclusions

A number of factors repeatedly surface in papers published across several continents, over a timespan of more than 20 years. Themes of intimidation, of exclusion, of lack of preparation and confused learning objectives are apparent in multiple studies, and negatively influence the medical students’ experience. They would appear to be powerful enough motivators to influence students’ very attendance in theatre in the first place, as well as their long-term career aspirations. These have been replicated in a number of settings and cited by both undergraduate and postgraduate-entry medical students in their clinical years in medical schools internationally.

There is much to be learnt from the sentiments echoed by students throughout the literature on their experiences in theatre.  It is perhaps disheartening that so many of the obstacles they encountered were avoidable.  Yet for this very reason, so too can we be inspired.  We feel that it is a very achievable feat for educators in the surgical context to modify many of the variables outlined above, and thus we are empowered to improve the theatre experience of our future medical students.

### Conflict of Interest

The authors declare that they have no conflict of interest.
